# Progesterone is inversely associated with optic nerve sheath diameter in dogs: a cross-sectional analysis

**DOI:** 10.1007/s11259-026-11318-w

**Published:** 2026-06-06

**Authors:** Candemir Özcan, Ümran Akın Özcan, Kenan Çağrı Tümer, Tarik Safak

**Affiliations:** 1 Faculty of Veterinary Medicine, Department of Surgery, Kastamonu University, Kastamonu, Türkiye; 2 Faculty of Veterinary Medicine, Department of Internal Medicine, Kastamonu University, Kastamonu, Türkiye; 3 Faculty of Veterinary Medicine, Department of Obstetrics and Gynecology, Kastamonu University, Kastamonu, Türkiye

**Keywords:** Optic nerve sheath diameter, Dogs, Ultrasonography, Estradiol, Progesterone, Intracranial pressure, Body mass

## Abstract

Optic nerve sheath diameter (ONSD) is increasingly used as a non-invasive indicator of intracranial pressure and neurological status in both human and veterinary medicine, although physiological factors affecting ONSD measurements in dogs remain insufficiently defined. This study investigated the relationships among body mass (BM), age, serum estradiol (E2) and progesterone (P4) levels, and ultrasonographically measured ONSD in clinically healthy female dogs. Thirty-four female dogs of different ages and BM presented for routine clinical examination were included. ONSD was measured ultrasonographically via a transorbital approach using a microconvex probe, and the mean value of bilateral measurements was used for analysis. Serum E2 and P4 concentrations were determined using ELISA. Correlation analyses (Pearson or Spearman, depending on distribution) and power-law regression models were applied to evaluate associations between variables. Age and E2 were expressed as median (IQR) values [2.5 (1) years and 90.43 (85.03)], whereas BM, P4, and ONSD were reported as mean ± SD (23.15 ± 9.72 kg, 9.04 ± 1.96 ng/mL, and 2.42 ± 0.44 mm, respectively). A moderate negative correlation was observed between P4 and ONSD (*r* = − 0.41, *p* < 0.05), while no significant relationships were detected between age, BM, E2, and ONSD (*p* > 0.05). These findings suggest that P4 may show a modest inverse association with ONSD in clinically healthy female dogs, whereas BM, age, and E2 appear to have minimal influence. Further studies with larger populations are warranted to confirm these findings.

## Introduction

The optic nerve sheath diameter (ONSD) has emerged as a valuable, non-invasive marker for assessing intracranial pressure and neurological status in dogs (Valerio-López et al. 2025). Because the subarachnoid space surrounding the optic nerve is continuous with the intracranial compartment, changes in intracranial pressure can be reflected by variations in ONSD (Ilie et al. 2015). Ultrasonographic evaluation of ONSD is therefore considered a rapid, safe, and repeatable technique that can be performed bedside without the need for advanced imaging modalities (Valerio‐López et al., 2025).

In veterinary medicine, interest in ocular ultrasonography has increased substantially in recent years, particularly for the evaluation of orbital and neurological conditions. However, physiological factors that may influence ONSD measurements in dogs are not yet fully understood (Dupanloup and Osinchuk 2021). Variables such as body mass (BM), age, and systemic physiological status may affect ocular structures and vascular dynamics, potentially contributing to variability in ONSD values even in clinically healthy animals (Smith et al. 2018).

Sex steroid hormones, particularly estradiol (E2) and progesterone (P4), play critical roles in vascular regulation, fluid homeostasis, and neurophysiological function. Evidence from ophthalmologic research indicates that hormonal fluctuations may influence visual function, ocular perfusion, and intraocular pressure, suggesting potential effects on optic nerve physiology (Yucel et al. 2005). However, the possible association between circulating sex hormones and optic nerve sheath morphology remains poorly understood, particularly in veterinary medicine, where evidence is still limited.

Therefore, the aim of the present study was to investigate the relationship between BM, serum sex hormone levels (E2 and P4), and ultrasonographically measured ONSD in clinically healthy dogs. A better understanding of these physiological influences may help refine the interpretation of ONSD measurements and improve their clinical utility in veterinary neurology and ophthalmology.

## Materials and methods

### Animal

The study was conducted with the approval of the Kastamonu University Local Ethics Committee for Animal Experiments (Approval No: 2026/17; Date: 16.02.2026). A total of 34 clinically healthy female mixed-breed dogs of different ages and body weights presented for routine examination were included in the study. All animals underwent general physical and ophthalmologic examinations prior to inclusion, and only dogs without systemic or ocular pathology were enrolled. All procedures were non-invasive, performed with minimal restraint to reduce stress, and informed owner consent was obtained before sample collection and ultrasonographic evaluation.

### Ultrasound measurement of the optic nerve sheath diameter

**Fig. 1:**
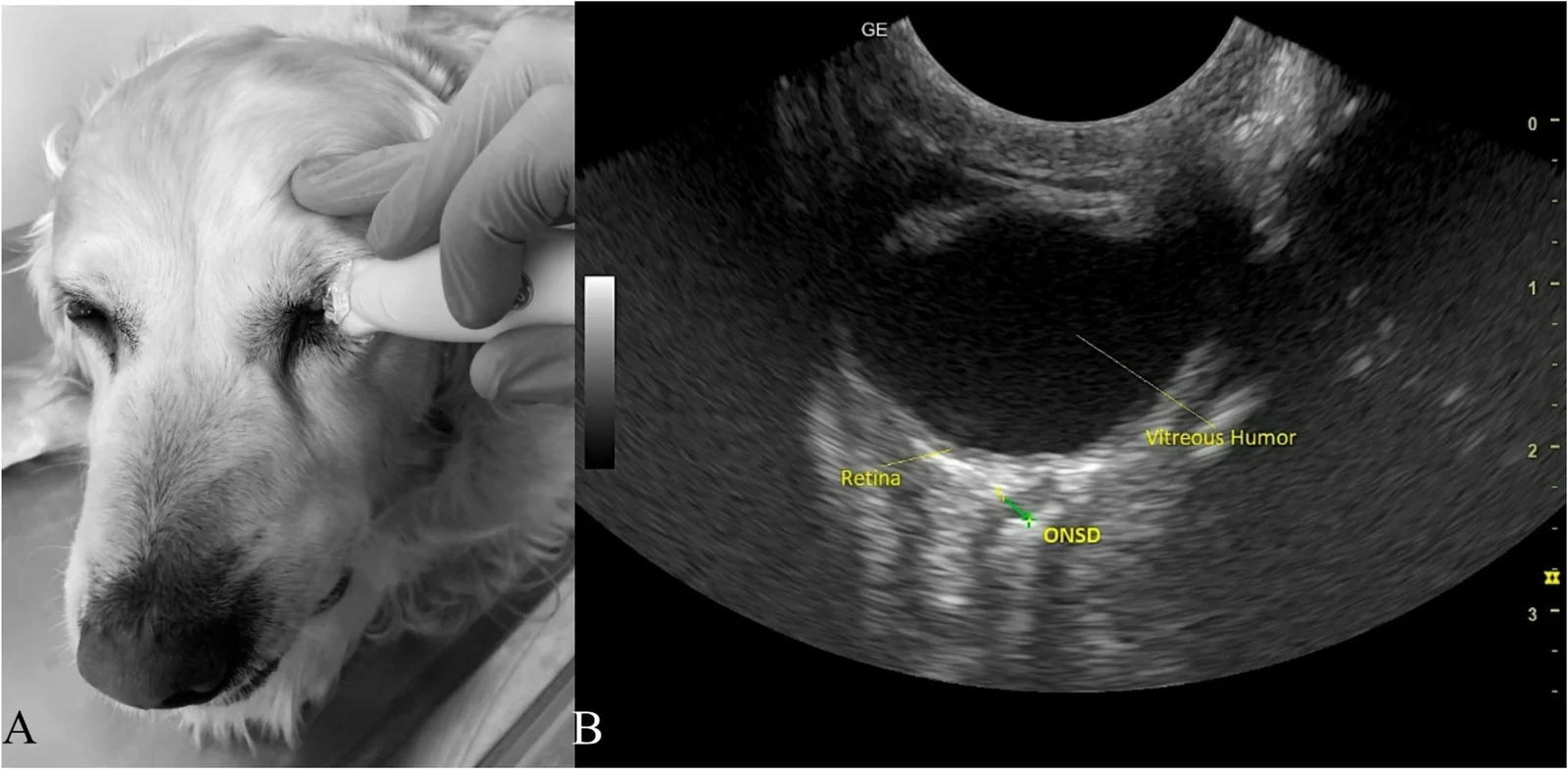
Ultrasonographic evaluation of optic nerve sheath diameter (ONSD) using a transorbital approach in dogs. (**A**) Positioning of the microconvex transducer over the closed upper eyelid for axial optic nerve imaging. (**B**) ONSD measurement performed approximately 3 mm posterior to the optic disc, perpendicular to the optic nerve axis

Examinations were performed via a transorbital approach with the animals in a calm resting position. Acoustic coupling gel was applied to the closed upper eyelid, and care was taken to avoid excessive pressure on the globe. The probe was gently positioned over the eyelid to obtain a clear axial image of the optic nerve (Fig. 1A).

In accordance with Özcan et al. (2025), ONSD was evaluated ultrasonographically using a Versana Active ultrasound system (General Electric, USA) equipped with an 8 C-RS microconvex transducer (4–10 MHz; General Electric, USA). This transducer was operated at its maximum frequency range to ensure high axial and lateral resolution, which is essential for the precise identification of superficial structures like the optic nerve sheath.

ONSD measurements were obtained approximately 3 mm posterior to the optic disc, perpendicular to the optic nerve axis. To ensure data reliability and eliminate inter-observer bias, all assessments were carried out by a single experienced observer. Measurements were performed in triplicate for both the right and left eyes; the mean value of these recordings was then calculated for each eye and used for final statistical analysis (Fig. 1B).

### Hormon analysis

Venous blood samples were collected from dogs for hormone analysis into plain biochemical tubes without anticoagulant. After allowing the blood to clot at room temperature, samples were centrifuged at approximately 3000 × g for 10 min to separate the serum. The obtained serum samples were properly labeled and stored at -20 °C until analysis. Serum E2 and P4 concentrations were measured using an enzyme-linked immunosorbent assay (ELISA) according to the manufacturer’s instructions. After all steps were completed, E2 and P4 levels were de termined by reading the plates at 450 nm in an ELISA reader (Bio Tek Instruments, USA) (Safak and Risvanli 2022).

### Statistical analysis

All statistical analyses were performed using SPSS software (IBM SPSS Statistics for Windows, Version 25.0). Correlation analyses were performed to evaluate relationships among variables. Pearson correlation (r) was used for variables showing approximately normal distribution, whereas Spearman rank correlation (ρ) was applied to non-normally distributed data. Correlation strength was interpreted according to coefficient magnitude, and statistical significance was assessed at conventional confidence levels. Normality assessment based on skewness–kurtosis values and central tendency measures indicated that BM, P4, and ONSD showed approximately normal distributions and were suitable for parametric analyses. In contrast, age and E2 displayed notable skewness and deviation from normality, suggesting that non-parametric analyses were more appropriate for these variables. Normally distributed data were expressed as mean ± standard deviation (SD), whereas non-normally distributed variables were presented as median (interquartile range, IQR). The relationship between BM, E2, P4 and ONSD was analyzed using a power-law model of the form:

$$ONSD=a+b\times BM^c$$

where 𝑎 represents the intercept, 𝑏 the scaling coefficient, and 𝑐 the exponent determining the curvature of the relationship. This model was preferred to capture potential non-linear biological scaling effects not addressed by linear regression.

## Results

**Fig. 2:**
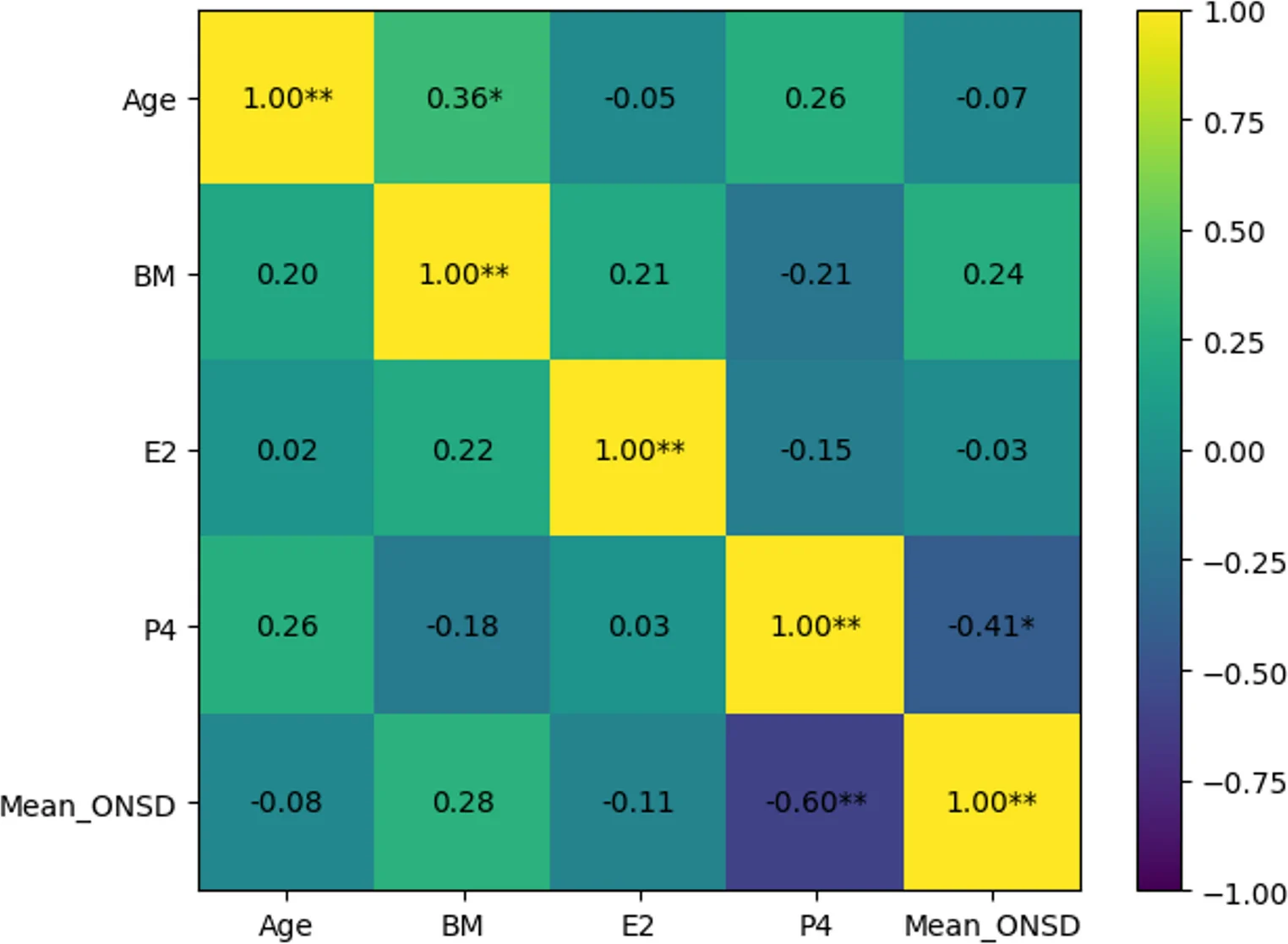
Heatmap illustrating Pearson (upper triangle) and Spearman (lower triangle) correlations among age, body mass (BM), estradiol (E2), progesterone (P4), and mean optic nerve sheath diameter (ONSD). Values within the cells denote correlation coefficients; statistical significance is indicated as ^*^ for *p* < 0.05 and ^**^ for *p* < 0.01

A total of 34 female dogs were included in the analysis. Age and E2 were presented as median (interquartile range), with values of 2.5 years (IQR: 1) and 90.43 pg/mL (IQR: 85.03), respectively. BM, P4, and ONSD were expressed as mean ± standard deviation, with values of 23.15 ± 9.72 kg, 9.04 ± 1.96 ng/mL, and 2.42 ± 0.44 mm, respectively. The relationships among age, BM, E2, P4, and ONSD were evaluated using Pearson and Spearman correlation analyses. According to the distribution characteristics of the variables, Pearson correlation (r) was used for parametric data and Spearman rank correlation (ρ) for non-parametric data. A moderate negative correlation was observed between P4 and ONSD in both analyses (*r* = -0.41, *p* < 0.05). No statistically significant correlations were detected between age (ρ = -0.08, *p* > 0.05), BM (*r* = 0.24, *p* > 0.05), E2 (ρ = -0.11, *p* > 0.05) levels, and ONSD in either Pearson or Spearman analyses (*p* > 0.05). Overall, apart from the inverse association between P4 levels and ONSD (*r* = -0.41, *p* < 0.05), the remaining variables showed weak or negligible correlations without statistical significance (Fig. 2).

The relationship between BM and ONSD was examined using a power-law model. The resulting best-fit curve was expressed as:

$$ONSD=-581.03+582.56\times BM^{0.00054}(R^2=0.08)$$

The model demonstrated a low explanatory power (𝑅^2^ = 0.08), indicating that BM accounted for only a small fraction of the variability in ONSD. The predicted ONSD values were 2.42 mm (range: 1.83–2.74 mm, 95% CI). The relationship between sex hormone levels and ONSD was evaluated using a power-law model. For E2, the best-fitting curve was expressed as:

$$ONSD=8.84-6.29\times E2^{0.0048}\;(R^2=0.001)$$

indicating virtually no relationship between E2 and ONSD, with predicted values 2.38 mm (range: 2.26–2.51 mm, 95% CI). For P4, the fitted model was:

$$ONSD=1.82+17.44\times P4^{-1.57}\;(R^2=0.23).$$

**Fig. 3:**
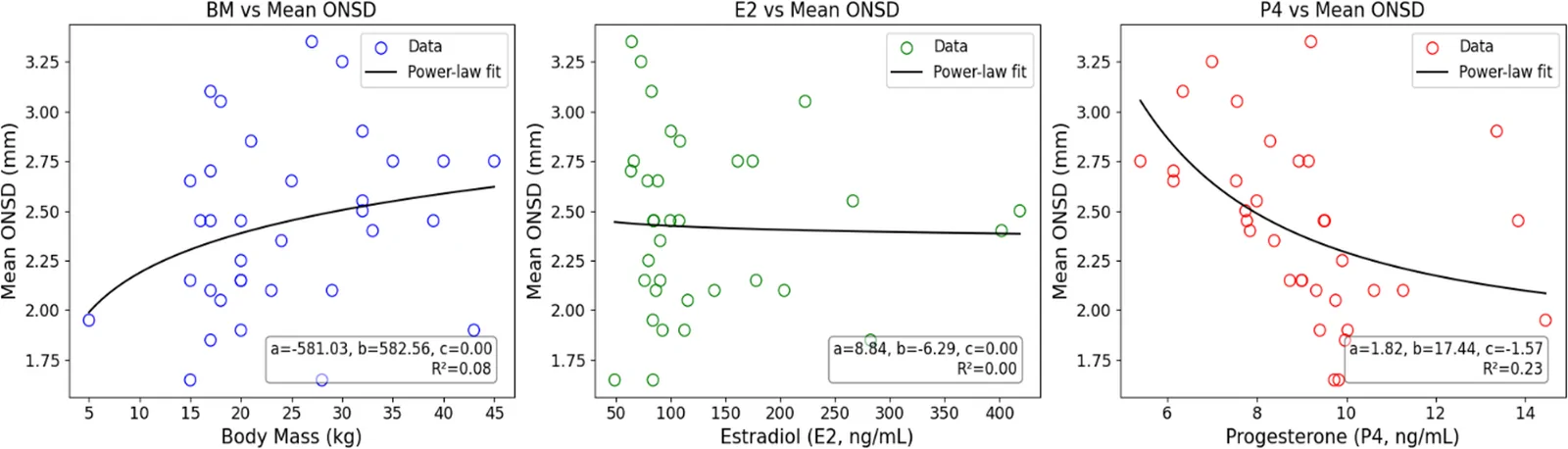
Power-law relationships between BM, sex hormone levels (E2, P4), and ONSD in dogs

This suggests a weak-to-moderate inverse association, with predicted ONSD values 2.42 mm (range: 1.71–3.15 mm, 95% CI). Scatter plots with the corresponding power-law fits are shown in Fig. 3.

## Discussion

In this study, the relationship between sex hormone levels, BM, and ultrasonographically measured ONSD in dogs was investigated. Our results revealed a moderate negative correlation between P4 and ONSD, while no significant associations were observed for age, BM, or E2. Power-law modeling suggested weak-to-moderate inverse relationship between P4 and ONSD (R² = 0.23), consistent with the correlation analysis, whereas BM and E2 accounted for minimal variance in ONSD (R² = 0.08 and 0.001, respectively).

These findings align with previous research and suggest that ONSD may be subtly influenced by hormonal factors, particularly P4. Özcan et al. (2025) demonstrated a significant effect of cerebrospinal fluid E2 on ONSD in female dogs, although our study did not find a meaningful relationship with E2. The discrepancy may be attributed to differences in sample size, hormonal phase variability, or measurement techniques. Similarly, Valerio-Lopez et al. (2025) highlighted that ONSD decreases following hyperosmolar therapy in dogs with presumed intracranial hypertension, supporting the concept that ONSD is a dynamic biomarker responsive to physiological and pharmacologic changes. Although our study did not involve therapeutic interventions, the negative association with P4 may reflect a subtle hormonal modulation of optic nerve sheath compliance.

In agreement with human and veterinary literature, we observed that BM had minimal influence on ONSD. Smith et al. (2018) reported that ONSD in healthy dogs was strongly associated with weight, yet the variance explained by BM was largely overshadowed by other factors, consistent with our low R² value in the BM–ONSD power-law model. Dupanloup and Osinchuk (2021) further confirmed that the ONSD to eyeball transverse diameter (ETD) ratio was consistent across dog breeds and morphologies, indicating that breed and body size minimally affect ONSD, which supports the robustness of our findings across a heterogenous cohort.

Methodological considerations are also important. Our use of transpalpebral ultrasonography parallels the approaches described by Valerio-Lopez et al. (2025), Smith et al. (2018), and Lee et al. (2003), all demonstrating excellent intra- and interobserver reliability. These results supports the reliability of ONSD measurement as a noninvasive biomarker in both research and clinical settings. Similarly, Evangelisti et al. (2020) confirmed repeatability of ONSD measurement in cats, supporting the general applicability of ultrasonographic ONSD assessment across species.

Additionally, high-resolution imaging studies provide anatomical context for interpreting ONSD variations. Ivan et al. (2022) demonstrated that 3T MRI allowed detailed visualization of the optic nerve sheath and ocular structures, with superior precision compared to ultrasonography. While MRI is considered the anatomical gold standard for precise orbital imaging, ultrasonography has established itself as the clinical gold standard for bedside monitoring due to its accessibility. Nevertheless, even if US-based measurements slightly underestimate absolute dimensions compared to MRI, the consistency of relative changes and correlations observed in our study remains valid. Moreover, Ilie et al. (2015) confirmed a positive, nonlinear relationship between acute increases in intracranial pressure (ICP) and ONSD in dogs, reinforcing that ONSD is a sensitive indicator of intracranial dynamics, even in healthy subjects. Similarly, Sahay et al. (2018) showed that pharmacologic modulation with dexmedetomidine attenuated ONSD increases during laparoscopic surgery, highlighting the influence of physiological and therapeutic factors on ONSD.

Finally, breed and sex differences may play a minor role. Lee et al. (2003) reported small but significant differences in ONSD among breeds, though sex, age, and body weight generally did not influence ONSD significantly. This is consistent with our finding that age, BM, and E2 were not significant predictors, while hormonal influences, particularly P4, may exert a subtle modulatory effect.

This study has several important limitations that should be considered when interpreting the findings. The sample size was relatively small, and only female dogs were included, which may limit the generalizability of the results. Although ONSD measurements are a widely used non-invasive surrogate for intracranial pressure, they are operator-dependent, and no direct intracranial pressure measurements were performed, which limits definitive conclusions regarding intracranial dynamics. Additionally, the cross-sectional design precludes establishing causal relationships between P4 levels and ONSD. Furthermore, the use of the ELISA method instead of Electrochemiluminescence Immunoassay (ECLIA) is a limitation, as ECLIA offers higher sensitivity and a broader dynamic range for hormonal analysis. Other physiological or environmental factors, such as hydration status, blood pressure, or stress levels, were not systematically controlled, which may introduce additional variability. By explicitly acknowledging these factors, the study provides a transparent framework for interpreting the results and highlights areas for future research.

In summary, our findings indicate that in healthy dogs, ONSD was not significantly associated with BM, age, or E2 levels, and may be moderately inversely associated with P4. These results support the potential of ONSD as a noninvasive marker of optic nerve sheath morphology in healthy dogs, while also suggesting that sex hormones, particularly P4, could exert a subtle modulatory effect. Future studies should explore the mechanistic basis of hormonal modulation of ONSD and its implications in clinical and physiologic contexts. While MRI remains the anatomical gold standard for further validation, ultrasonography offers a more practical and non-invasive alternative for real-time clinical assessment.

## Data Availability

The data that support the findings of this study are available from the corresponding author, upon reasonable request.
